# ShenShuai II Recipe Attenuates Apoptosis and Renal Fibrosis in Chronic Kidney Disease by Increasing Renal Blood Flow and Improving Oxygen Consumption

**DOI:** 10.1155/2018/7602962

**Published:** 2018-12-13

**Authors:** Meng Wang, Jing Yang, Yuan Zhou, Chen Wang

**Affiliations:** ^1^Department of Nephrology, Shuguang Hospital Affiliated to Shanghai University of Traditional Chinese Medicine, Shanghai 201203, China; ^2^Key Laboratory of Liver and Kidney Diseases, Chinese Ministry of Education, Shanghai University of Traditional Chinese Medicine, Shanghai 201203, China; ^3^TCM Institute of Kidney Disease, Shanghai University of Traditional Chinese Medicine, Shanghai 201203, China

## Abstract

*Background*. Hypoxia plays a significant role in the progression of chronic kidney disease (CKD) and renal fibrosis. In China, Chinese herbal medicine has been widely used to treat CKD. ShenShuai II Recipe (SSR) is a commonly used prescription which has shown good results against CKD. However, the exact mechanisms of SSR are still unknown. In this study, chronic renal failure (CRF) was induced in rats by the 5/6 renal ablation/infarction (A/I) surgery; we investigated the efficacy and mechanisms of SSR on CKD in the current study. Male Sprague-Dawley (SD) rats were divided into the four groups: (1) sham operation group, (2) 5/6 (A/I) model group, (3) 5/6 (A/I) +SSR group, and (4) 5/6 (A/I) +Losartan group (5/6 (A/I) +Los). After 8 weeks of treatment, we evaluated renal blood flow (RBF) and oxygen consumption along with renal function, apoptosis, and renal fibrosis. Our results showed that SSR significantly improved RBF and reduced intrarenal oxygen consumption and apoptosis. Moreover, SSR markedly attenuated interstitial fibrosis, accompanied by decreased levels of serum creatinine (Scr), serum uric acid (UA), increased hemoglobin (HB), and evaluated glomerular filtration rates (eGFRs). These results suggest that SSR could mediate renal protection by improving intrarenal hypoxia and, furthermore, participate in the antiapoptotic effects by downregulating apoptosis markers (cleaved caspase-3 and the ratio of Bax/Bcl2) in 5/6 (A/I) model with CRF rats.

## 1. Introduction

Chronic kidney disease (CKD) is a common concern associated with high mortality and disability worldwide, which leads to much lower quality of life and a substantial economic burden [1, 2]. Renal fibrosis is main pathological basis for the progression of CKD to end-stage renal disease [3]. The activation of interstitial fibroblasts and the deposition of extracellular matrix (ECM) components are involved in the formation of renal interstitial fibrosis, which results in destruction of the renal architecture, progressive decrease of renal function [4]. With the in-depth studies on the pathogenesis of renal fibrosis, hypoxia plays an important role in promoting CKD progression and fibrosis [5]. We confirmed that renal fibrosis originated from the increase of remnant nephron oxygen consumption, resulting in intrarenal hypoxia [6]. Kidneys account for 0.4%-0.5% of human body weight, but oxygen consumption accounts for about 7% of human body. This physiological characteristic of high demand for oxygen leads to easy oxygen deficiency in kidney diseases [7].

In hypoxia, hypoxia inducible factor prolyl hydroxylase domain protein is inactive since it requires molecular oxygen for its activity [8]. Therefore, HIF-1*α*, a core transcription factor under hypoxic conditions, can be stable expression. HIF-1*α* also transcribes apoptosis-related genes to trigger apoptosis [9]. Previous studies confirmed that apoptosis was closely associated with renal fibrosis. Zhou Jun et al. reported that TAK1 promoted renal fibrosis by regulating P38-induced apoptosis [10]. Xiangjun et al. confirmed that puerarin could attenuate renal fibrosis by reducing epithelial cell apoptosis [11]. Therefore, inhibition of apoptosis could be an effective measure to reduce renal fibrosis.

Traditional Chinese Medicine (TCM) has a long history in treating CKD in China. In the clinic, we observed that ShenShuai II Recipe (SSR) could improve renal function and anemia, increase the levels of hemoglobin (HB) and red blood cells (RBC). However, the specific molecular mechanisms are unclear. Therefore, based on the evidences, this study was performed to investigate whether SSR could attenuate apoptosis and renal fibrosis by improving renal hemodynamics by means of 5/6 renal ablation/infarction(A/I) model (typical intrarenal hypoxia model [6, 12]). Expanding our understanding of drug mechanisms will provide useful strategy for the clinic.

## 2. Materials and Methods

### 2.1. Animals and Drugs

Male Sprague-Dawley (SD) rats weighing 190-210 g were purchased from Shanghai SLAC Laboratory Animal Co., Ltd. SSR consists of nine herbs,* Codonopsis pilosula 15g, Epimedium 15g, Salvia miltiorrhiza bge 15g, Angelica sinensis 15g, Rheum palmatum L 15g, Coptis chinensis 6g, Folium perillae 15g, Ligusticum chuanxiong 15g, Peach kernel 15g, and total 126g. *All were purchased from Shuguang Hospital and identified by Department of TCMs. The above nine herbs were mixed and boiled twice with water; the liquid medicine was merged and concentrated. In the end, the liquid medicine was concentrated to about 21mL and gavage dose was 10mL/kg. Losartan was purchased from Merck Sharp & Dohme (Hang zhou, China) and made into 5mg/mL liquid medicine with deionized water. The gavage dose was 6mL/kg.

### 2.2. Rat CKD Model

All experimental procedures were approved by the Animal Ethics Committee of Shanghai University of TCM. CKD was induced in rats by 5/6 (A/I) as previously described [6]. Briefly, The 5/6 (A/I) operation was performed in rats under anesthesia with sodium pentobarbital (40mg/kg body weight, i.p.) by ligation of two branches of the left renal artery. After one week, the right kidney was removal. The rats were kept warm in an incubator until fully ambulatory.

### 2.3. The Animal Study Protocol

Four weeks after 5/6 A/I, 45 rats were randomized into 3 groups, one received saline treatment, one received losartan treatment (6mL/kg daily by gavage) and another received SSR treatment (10mL/kg daily by gavage). A group of 15 rats that received sham operation were also included in the study. Metabolic cage collected 24h total urine, renal function, and blood routine was measured before and at the end of 8-week treatment. Kidney tissues were harvested at necropsy for histology studies and molecular assessment.

### 2.4. Renal Function and Oxygen Consumption Measurement

Serum creatinine (Scr), uric acid (UA), and hemoglobin (HB) were detected by Automatic biochemical analyzer. Instead of GFR, Ccr is calculated as follows: Ccr (ml/min) = urine creatinine × 24h urine volume (ml)/serum creatinine × 1440.

The oxygen consumption was measured under anesthesia with sodium pentobarbital (40mg/kg body weight, i.p.). The left kidney blood flow (ml/min) was continuously recorded using a flow probe (Transonics T420, USA) which was linked to a computer. Blood samples, which were collected from the abdominal aorta and proximal left renal vein, were used to measure total arterial blood hemoglobin (tHb), (O2Hb), (pO2), (pCO2), pH, [Na+], [K+], and [HCO3-] with the blood gas analyzer and biochemical multiple test cards(i-STAT EG7, USA, Abbott). O_2_ content (O_2_ct, ml/ml blood) = (1.39 xtHbxO_2_Hb% + pO_2_ x 0.003)÷100. The total left kidney O_2_ consumption (QO_2_, ml/min) = (A-V difference in O_2_ct) × renal blood flow (RBF); TNa is equal to the total amount of sodium filtered (FNa) minus the amount of sodium excreted in the urine (UNaV).

### 2.5. Western Blot

The protein concentration was calculated by the Bradford method. Proteins were separated by 8% or 12% gel and were electro-transferred to a polyvinylidene difluoride membrane (Merck). The membrane was incubated in the blocking buffer (5% nonfat milk, 20mM Tris-HCl, 150mMNaCl, pH 8.0, 0.01%Tween 20) for 1 hour and was followed by incubation with anti-HIF-1*α* (A11945 Abclonal), anti-nNOS (4231 CST), anti-Bax (AB32503 Abcam), anti-caspase-3 (9662S CST), anti-Bcl-2 (GXP47121 Genspan), anti-fibronectin (AB23750 Abcam), anti-*α*-SMA (AB5694 Abcam), and anti-collagen-I (AB6308 Abcam) overnight at 4°C. Next, the membrane was incubated with secondary antibodies (1:1000, Proteintech) for 1 hour. The signal was detected by an enhanced chemiluminescence kit (BeyoECL Star, P0018A, Byotime). Quantitative analysis was performed using Quantity One Analyzer (Bio-Rad).

### 2.6. Histopathological Examinations and Immunohistochemical (IHC) Staining

After embedding kidney tissue into paraffin, three-micrometer-thick sections were used for Masson's trichrome staining according to the standard protocol. For immunohistochemistry staining, after antigen retrieval (100×Citrate solution, Sangon biotech, Shanghai, China) and blocking the endogenous peroxidase activity, the sections were blocked and then incubated with anti-HIF-1*ɑ* (1:200, Abcam) and anti-cleaved caspase-3 (1:200, CST) overnight at 4°C. The sections were rinsed in PBS 3 times and then incubated with biotinylated goat anti-rabbit IgG. Positive staining was characterized as brown using the DAB staining kit (Sangon biotech, Shanghai, China) and observed by bright field microscopy (Nikon Eclipse 80i, Japan). Four areas were randomly selected in each section and examined at 200× magnification. Pictures were analyzed by ImagePro plus version 6.0.

### 2.7. TUNEL Assay

One-step TUNEL assay kit (Beyotime, Shanghai, China) was used to detect apoptosis according to the standard protocol. Paraffin-embedded sections were incubated with proteinase K for 30min at 37°C. Next, each slice was incubated with 50*μ*L TUNEL mixture for 1 h at 37°C in the dark. The cells with green fluorescence were defined as apoptotic cells using fluorescence microscopy (Nikon Eclipse 80i, Japan) at 200× magnification. At least 4 areas were selected for each slide.

### 2.8. Statistical Analysis

All data were presented as mean ± SE. Data were analyzed by two-tailed paired Student's t-test or one-way analysis of variance (ANOVA) with LSD-t's multiple comparisons, using statistical software SPSS 18.0 (SPSS Ltd., Chicago, IL, USA). P<0.05 is considered statistically significant.

## 3. Results

### 3.1. ShenShuai II Recipe Improved Renal Function, Ameliorated Interstitial Fibrosis, and Inhibited Expression Levels of Fibronectin (FN), Collagen-I(Col-I), and *α*-Smooth Muscle Actin (*ɑ*-SMA) Protein in the Remnant Kidneys with CRF

**Figure 1:**
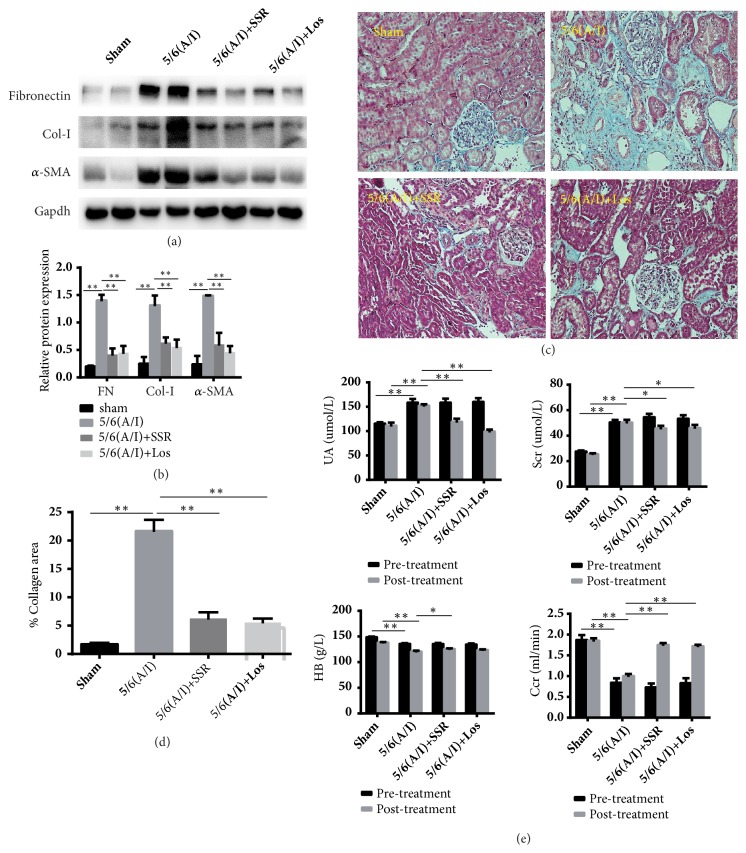
Effects of SSR on renal function and interstitial fibrosis in 5/6th (A/I) rats. (a) The protein levels of Col-I, FN, and *α*-SMA were detected by western blot. (b) The ratio of Col-I, FN, and *α*-SMA to GAPDH protein was calculated (n=6). (c) Representative photomicrographs of Masson staining. Original magnification, ×200. (d) Semiquantitative analysis of collagen area (n=4). (e) The levels of Scr, Ccr, HB, and UA were measured at 4 weeks and 12 weeks after operation (n=15). Values are mean ± SE. ^*∗*^P<0.05; ^*∗∗*^P<0.01.

Activation of renal fibroblasts and deposition of ECM play a predominant role in development and progression of renal fibrosis [4, 13, 14]. Therefore, we mainly examined expression of *α*-SMA Col-I and FN proteins. Western blot examination showed that the expression of *α*-SMA protein was increased and the analysis of Col-I and FN expression showed the same pattern as that of *α*-SMA in model group. SSR treatment markedly decreased *α*-SMA, Col-I, and FN expression at protein levels in 5/6 (A/I) rats. (Figures 1(a) and 1(b))

In addition, histopathological examinations showed significantly interstitial fibrosis in 5/6(A/I) group (Figures 1(c) and 1(d)). Blood biochemical test indicated UA, an early hallmark of renal dysfunction, markedly elevated in model group, while Scr, GFR and HB, which were considered as serious biochemical indicators in chronic renal failure, were significantly worse in 5/6(A/I) model group (Figure 1(e)). Conversely, SSR dramatically ameliorated interstitial fibrosis and improved renal biochemical indicators.

### 3.2. ShenShuai II Recipe Improved Renal Blood Flow (RBF) and Reduced Remnant Renal Oxygen Consumption in 5/6(A/I) Rats

**Figure 2:**
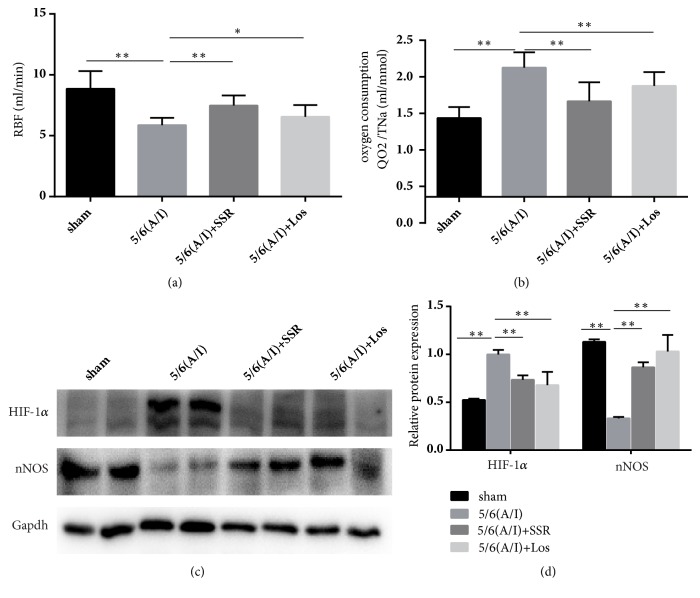
Effects of SSR on renal blood flow and oxygen consumption in the remnant kidney. (a) Renal blood flow was measured after treatment (n=15). (b) Total intrarenal oxygen consumption factored by QO2/TNa was tested after treatment (n=15). (c) The protein expression of HIF-1*α* and nNOS was determined by western blot. (d) The ratio of HIF-1*α* and nNOS to GAPDH protein was calculated (n=6). Values are mean ± SE. ^*∗*^P<0.05; ^*∗∗*^P<0.01.

Our data showed that RBF was significantly lower in the 5/6 (A/I) model group as compared to that in the sham group (5.87 ± 0.6 versus 8.85 ± 1.45 P<0.01 Figure 2(a)). Treatment with SSR increased RBF (7.84 ± 0.83 versus 5.87 ± 0.6 P<0.01 Figure 2(a)). Oxygen consumption, reflected by sodium transport efficiency (QO2/TNa), was increased by 5/6(A/I) (1.77 ± 0.21 versus 1.08 ± 0.15, ml/mmol, P<0.01 Figure 2(b)). QO2/TNa was significantly lower after 8 weeks treatment of SSR (1.31 ± 0.26 versus 1.77 ± 0.21, ml/mmol, p<0.01 Figure 2(b)). Furthermore, we determined the contents of nNos and HIF-1*α* protein, markers for oxygen consumption and hypoxia. As shown in Figures 2(c) and 2(d), the expression of HIF-1*α* protein was increased and the expression of nNOS protein was decreased in the 5/6 (A/I) group as compared to that in the sham-operated group. SSR treatment for 8 weeks decreased HIF-1*α* expression and increased nNOS expression at protein levels in 5/6 (A/I) rats.

### 3.3. ShenShuai II Recipe Produced the Antiapoptosis Effect by Regulating the Expressions of Apoptosis-Related Proteins in CRF Model

**Figure 3:**
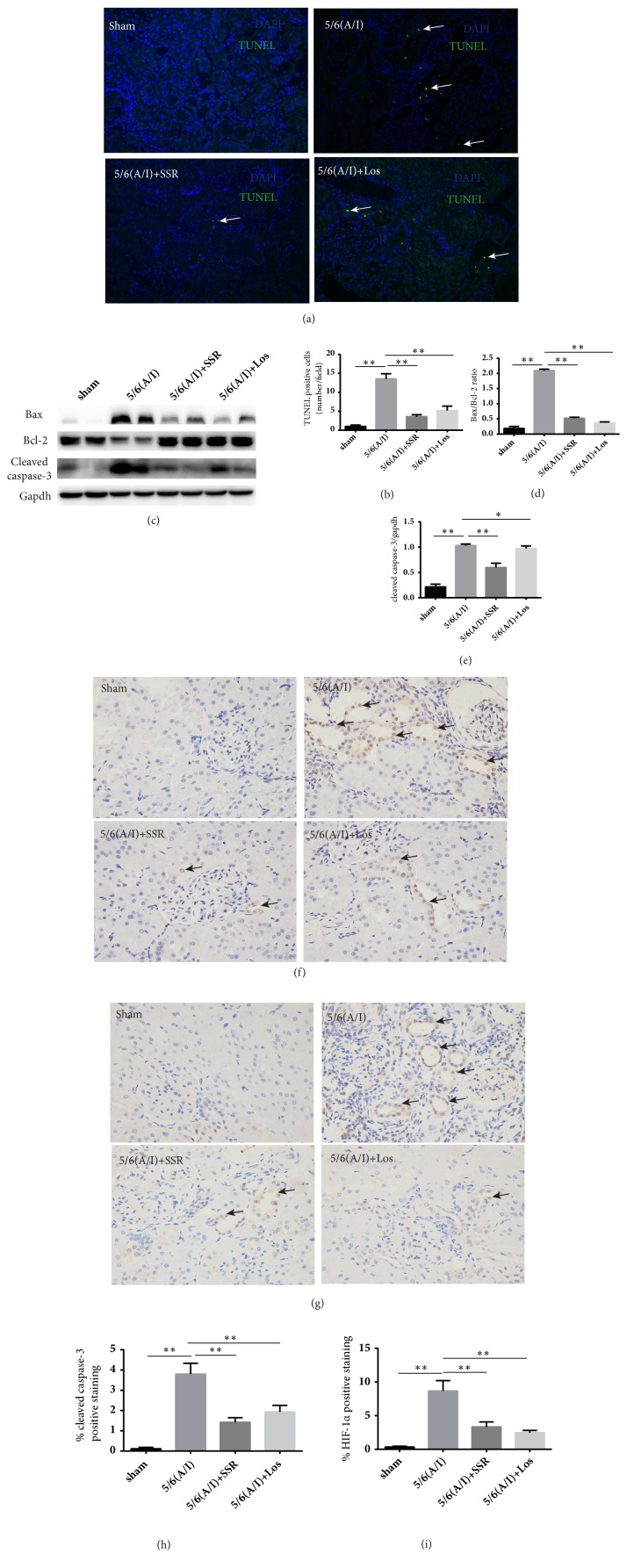
Effects of SSR on apoptosis in 5/6th (A/I) rats. (a) Representative image of TUNEL staining. Apoptotic cells are visualized as green (white arrow) and nuclei are stained with DAPI (blue). Images are shown at identical magnification, ×200. (b) Quantitative analysis for the numbers of TUNEL staining positive cells (n=4). (c) Protein expression of Bax, Bcl-2, and cleaved caspase-3 was determined by western blot. (d) The ratio of Bax to Bcl-2 protein was calculated (n=6). (e) The ratio of cleaved caspase-3 to GAPDH protein was calculated (n=6). (f) Representative image of IHC staining for cleaved caspase-3 (black arrow). Original magnification, ×200. (g) Representative image of IHC staining for HIF-1*ɑ* (black arrow). Original magnification, ×200. (h) Semiquantitative analysis of cleaved caspase-3 positive staining (n=4). (i) Semiquantitative analysis of HIF-1*α* positive staining (n=4). Values are mean ± SE. ^*∗*^P<0.05; ^*∗∗*^P<0.01.

TUNEL assay was used to examine condensed or fragmented nuclei of apoptotic cells. As shown in Figures 3(a) and 3(b), TUNEL green fluorescence (white arrow) presented that the numbers of apoptotic cells in 5/6(A/I) group in rats were markedly increased compared with sham-operated group. Conversely, SSR treatment significantly attenuated apoptosis induced by 5/6(A/I) operated kidneys. To determine the mechanism by which SSR reduces renal apoptosis after ischemic insult, we measured protein contents of Bax, Bcl-2, and cleaved caspase-3, hallmark of apoptosis. As shown in Figures 3(c), 3(d), and 3(e), western blot assay revealed that caspase-3 activation and the ratio of Bax to Bcl-2 protein dramatically increased in 5/6(A/I) model group. Meanwhile, treatment with SSR suppressed the activation of caspase-3 and decreased the ratio of Bax to Bcl-2 protein compared with model group. IHC staining confirmed HIF-1*α* and cleaved caspase-3 were mainly coexist in tubular cells (Figures 3(f) and 3(g)). Taken together, these results indicate that SSR could produce antiapoptosis effect on apoptosis-related proteins expression, which ultimately leads to decrease the ratio of Bax to Bcl-2 protein and caspase-3 activation.

## 4. Discussion

In this study, we found that SSR could significantly increase RBF and improve intrarenal hypoxia in the 5/6 (A/I) model of CRF in rats. Further, SSR could markedly alleviate the expression of HIF-1*α* protein, increase the expression of nNOS protein and produce antiapoptosis effect by attenuating apoptotic markers (cleaved caspase-3 and ratio of Bax/Bcl2). These changes are associated with improved renal function (Scr, UA, HB, and eGFR). These findings showed that SSR treatment for 8 weeks significantly inhibited ischemic insult-induced apoptosis and interstitial fibrosis in 5/6(A/I) rats.

Chronic hypoxia exacerbates renal fibrosis. The main regulator of hypoxia is HIF-1 and its oxygen-sensitive *α* subunit [15]. Although upregulation of HIF-1*α* has been shown to be protective in acute renal injury, increasing evidences suggest that continuous overactivation of HIF promotes progression of renal fibrosis [16–18]. Simultaneously, interstitial fibrosis could injury peritubular capillary formation and exacerbate hypoxia, forming a vicious cycle between interstitial fibrosis and hypoxia [15]. In our study, we found that remnant renal tissue blood flow decreased and oxygen consumption increased in the 5/6(A/I) model. We also observed elevated the levels of Scr, UA, and declined the levels of HB, eGFR, and severe interstitial fibrosis in 5/6(A/I) model group, which is further support of the harmfulness of hypoxia. Conversely, SSR dramatically decreased the levels of oxygen consumption factored by QO2/TNa and markedly increased RBF, HB, and eGFR, with decreased the levels of Scr and UA, verifying that one of the mechanisms by which SSR protects renal function and delays the progression of renal interstitial fibrosis is to improve intrarenal hypoxia.

Apoptosis is a process of programmed cell death, which is characterized by caspase activation, DNA ladder fragmentation, and formation of apoptosis bodies [19]. Apoptosis involves two important members of the Bc1-2 family, Bc1-2 and Bax. Bax interaction with the antiapoptotic protein Bcl-2 is a molecular switch that regulates the mitochondrial apoptosis pathway [20]. It has been reported that Bcl-2/Bax pathway was involved in Ang-II induced intestinal epithelial cells apoptosis [21]. Chen Hui et al. reported that emodin protects HK-2 cells from apoptosis after hypoxia/reoxygenation, accompanied by decrease of Bax/Bcl-2 ratio and caspase-3 activation [22]. Therefore, in our work, we investigated whether SSR could attenuate apoptosis by downregulating the ratio of Bax to Bcl-2 protein and caspase-3 activation. TUNEL assay were used to assess the degree of apoptosis. Our results revealed that SSR significantly lowered activation of caspase-3, decreased the ratio of Bax to Bcl-2 protein, and inhibited the cell apoptosis induced by hypoxia in 5/6(A/I) model. According to Brenner^,^s theory, kidney diseases mainly originate from glomerular hemodynamic changes, but increasing evidences showed that the degree of tubulointerstitial damage could be more closely associated with renal dysfunction. Therefore, the final common pathway of CRF operates principally in the tubulointerstitium [23–25]. Using IHC examination, we analyzed the expression position of cleaved caspase-3, a marker for apoptosis. Our data showed that cleaved caspase-3 mainly is located in renal tubular, where HIF-1*α* is predominantly expressed [26], which is consistent with previous studies.

In conclusion, we demonstrated that SSR could mediate renal protection by improving intrarenal hypoxia and, furthermore, participate in the antiapoptotic effects by downregulating apoptosis markers (cleaved caspase-3 and the ratio of Bax/Bcl2) in 5/6th (A/I) model with CRF rats.

## Data Availability

The data used to support the findings of this study are available from the corresponding author upon request.
